# Intradermal Electroporation of Naked Replicon RNA Elicits Strong Immune Responses

**DOI:** 10.1371/journal.pone.0029732

**Published:** 2012-01-04

**Authors:** Daniel X. Johansson, Karl Ljungberg, Maria Kakoulidou, Peter Liljeström

**Affiliations:** Department of Microbiology, Tumor and Cell Biology, Karolinska Institutet, Stockholm, Sweden; University Paris Sud, France

## Abstract

RNA-based vaccines represent an interesting immunization modality, but suffer from poor stability and a lack of efficient and clinically feasible delivery technologies. This study evaluates the immunogenic potential of naked *in vitro* transcribed Semliki Forest virus replicon RNA (RREP) delivered intradermally in combination with electroporation. Replicon-immunized mice showed a strong cellular and humoral response, contrary to mice immunized with regular mRNA. RREP-elicited induction of interferon-γ secreting CD8+ T cells and antibody responses were significantly increased by electroporation. CD8+ T cell responses remained substantial five weeks post vaccination, and antigen-specific CD8+ T cells with phenotypic characteristics of both effector and central memory cells were identified. The immune response during the contraction phase was further increased by a booster immunization, and the proportion of effector memory cells increased significantly. These results demonstrate that naked RREP delivered via intradermal electroporation constitute an immunogenic, safe and attractive alternative immunization strategy to DNA-based vaccines.

## Introduction

Since its introduction in the early 1990s, nucleic acid-based vaccination has emerged as a promising approach to elicit both cellular and humoral immune responses [1], [2]. Major advantages include relatively low production cost, high stability, ease of manipulation and the possibility to express complex antigens such as transmembrane proteins.

Although most focus has been on plasmid-based DNA vaccines, the use of RNA has advantages. For instance, the theoretical risk of vector integration into the host genome and subsequent malignant cell transformation is omitted. Due to the relatively short half-life of the RNA molecule, expression is transient. This decreases the risk when using tumor-associated antigen genes such as proto-oncogenes for immunization. In addition, RNA-based therapeutics is not classified as gene therapy by regulatory authorities, facilitating a more rapid advance into clinical trials of vaccine candidates.

The use of both naked and liposome-encapsulated mRNA has been validated in animal models for induction of antibodies and cytotoxic T lymphocytes (CTL) targeting cancer and infectious diseases [3], [4], [5], [6], [7]. Vaccination of cancer patients in two Phase I Clinical trials also demonstrated safety as well as increased cellular or humoral immunity in some patients, respectively [8], [9]. However, mRNA-elicited immune responses have often been weak and required multiple immunizations. Thus far, perhaps the most promising form of RNA vaccination is based on *ex vivo* tumor antigen-transfected autologous bone marrow-derived dendritic cells (DC) that are readminstered to the patient (reviewed in [10]). This approach has demonstrated induction of immunological responses in clinical trials with cancer patients and has in some cases been associated with tumor regression [11]. Albeit an attractive therapeutic avenue, personalized vaccines are not the path towards prophylactic immunization of the masses. Preventive vaccination requires fast and reliable administration in the field, without the need for complex medical infrastructure.

We have previously developed suicidal viral vectors, DNA and naked RNA vectors based on the alphavirus Semliki Forest virus (SFV) replicon [12], [13], [14], [15]. Upon transfection and nuclear localization, the DNA launched replicon (DREP) is transcribed from a Cytomegalovirus (CMV) promoter and exported to the cytoplasm. Once in the cytoplasm, the DREP, viral particle delivered replicon and naked RNA replicon (RREP) amplification steps are identical (described in more detail in [16]). First, the 5′ two thirds of the genome encoding the four replicase genes is translated. The replicase complex amplifies the genomic RNA and later transcribes large amounts of antigen-encoding mRNA from the 26S subgenomic viral promoter located downstream of the replicase genes. In addition to high expression levels of the inserted antigen encoding gene, the various RNA-species produced by the replicon amplification provide potent immunostimulatory ligands to pattern recognition receptors (PRR) such as TLR3, PKR and MDA-5 [17], [18]. The antiviral program initiated by replicon amplification and PRR signaling results in type I interferon production and induces apoptosis [19], [20], [21], thereby promoting cross-priming of antigen epitopes on MHC class I [22]. In addition, alphavirus replicon RNA has an increased stability due to its secondary structure, which protects it from degradation [23]. Accordingly, the replicon design has proven to be highly immunogenic, typically only needing one immunization to elicit a strong immune response contrary to conventional nucleic acid-based vaccines [12], [13], [14].

In a previous study, we have delivered a DNA launched replicon intradermally by needle injection, inducing a potent immune response [12]. The skin has a relatively high proportion of professional antigen presenting cells such as Langerhans cells and skin-resident DC, thus offering an attractive target tissue for immunization. *In vivo* electroporation is a technological advancement that has been used to augment *in vivo* transfection efficiency and subsequent gene expression from nucleic acids injected into the muscle [24], [25]. Contrary to intramuscular (i.m.) electroporation, intradermal (i.d.) electroporation is non-invasive, causes only minimal pain and is well tolerated [26], [27]. Currently, needle-free delivery methods are being developed further streamlining the use of this technology.

In this study, we investigated the potency of naked RNA to elicit an immune response by administering RNA replicon-based immunogens. We demonstrate that RREP, but not mRNA, is able to elicit both strong humoral and cellular immune responses that could be increased by electroporation. Thus, we present an alternative to mRNA and DNA-based vaccines with an improved safety and immunogenicity profile.

## Methods

### Plasmid construction, DNA and RNA preparation

DREP-tLuc, DREP-β-gal, pGEM-tLuc, pGEM-β-gal and pCMV-tLuc were produced by standard molecular cloning techniques. Plasmids were grown in *E.coli* and purified using the Endofree Plasmid Mega Kit (#12381, Qiagen GmbH, Hilden, Germany). RNA *in vitro* transcription was made from these plasmids using the mMessage mMachine SP6 (AM1340, Ambion, Invitrogen). All RNAs were purified using the RNeasy Mini Kit (#74104, Qiagen) prior to immunizations. All constructs used for immunizations are summarized in Table 1.

**Table 1:** 

Name	Description
mRNA-tLuc	*in vitro* transcribed mRNA encoding luciferase
RREP-tLuc	*in vitro* transcribed RNA replicon encoding luciferase
DREP-tLuc	DNA launched replicon encoding luciferase
pCMV-tLuc	DNA encoding luciferase under a CMV promoter
mRNA-β-gal	*in vitro* transcribed mRNA encoding β-galactosidase
RREP-β-gal	*in vitro* transcribed RNA replicon encoding β-galactosidase
DREP-β-gal	DNA launched replicon encoding β-galactosidase

### Mice and immunizations

C57BL/6 and 129sv/ew mice were bred and kept at the MTC animal facilities at Karolinska Institutet, Stockholm, Sweden, in accordance with the recommendations of the National Board for Laboratory Animals and used at the age of 6–9 weeks. The protocol was approved by the Northern Stockholm Board for Laboratory Animal Ethics, protocol number N374/08. Prior to immunization, mice were shaved on their lower back and anesthetized with 4% isoflurane. 20 µl of RNA or DNA diluted in PBS were injected intradermally (i.d.) to each flank in the lower part of the back followed by immediate electroporation (E.P.) with Derma Vax™ Clinical DNA Vaccine Delivery System (Cellectis SA, Romainville, France) at the injection sites. Electroporation consisted of 2 pulses of 1.125 V/cm for 50 µs, and 8 pulses of 275 V/cm for 10 ms. The needle-array electrodes (NE-4-4) with two parallel rows of four 2-mm pins (1.5×4 mm gaps) were used for electroporation.

### *In vivo* bioluminescence imaging

To monitor *in vivo* luciferase protein expression, 129sv/ew mice were injected i.p. with 1.5 mg D-luciferin (Caliper Life Sciences, Hopkinton, MA) diluted in PBS to a final volume of 100 µl and anesthetized with isoflurane. *In vivo* luciferase expression, measured as photonic emissions (photons/s/cm^2^) using an *in vivo* imaging system 100 (IVIS 100; Caliper Life Sciences), was performed 17 minutes after administration of luciferin (the signal peaked 17 minutes after injection, data not shown). Using the Living Image software (version 2.50.1; Caliper Life Sciences) image acquisition parameters were set at 10 seconds exposure time and medium binning. The intensity of the luminescence within the region of interest was quantified using the same software. Background luminescence was determined by measuring luminescence from naive mice. To calculate the integrated luciferase signal between two time points the following formula was used: (t_2_−t_1_)(s_2_+(s_1_−s_2_)/2) where t_1_ is timepoint 1, t_2_ timepoint 2, s_1_ signal at timepoint 1 and s_2_ signal at timepoint 2. To calculate the total accumulated signal, integrated signals from all time points were added up to the last time point of measurement (150 hours post immunization). These calculations are further clarified in Figure S1.

### Determination of antigen-specific IFN-γ secreting cells

Spleens were ground through a 70 µm cell grinder (Becton, Dickinson and company, Franklin Lakes, NJ) in a petri dish with 5 ml RPMI-1640 (Sigma-Aldrich Co, St Louis, Mo) supplemented with penicillin-streptomycin 100 units/ml (Gibco #15140, Invitrogen), L-glutamine 0.3 ml/ml (Gibco #25030, Invitrogen) and 5% heat inactivated FCS (Gibco #10270-106, Invitrogen). The cells were centrifuged at 400 g at room temperature for 7 minutes before they were resuspended in red cell lysis buffer (Sigma-Aldrich). Lysis was carried out for 2 minutes and the lymphocytes were subsequently washed and resuspended in RPMI-1640. Cells were seeded (200,000 per well) with 2 µg/ml SIINFEKL peptide (ProImmune Ltd, Oxford, United Kingdom) or 2.5 µg/ml ConA (Sigma-Aldrich Co) as a positive control in 96-well filter plates (Millipore) coated with anti-IFN-γ antibodies (Mabtech, Nacka, Sweden). Cells were then cultured for 20 hours at 37°C, 5% CO_2_ and developed as recommended by the manufacturer using biotinylated anti-IFN-γ, streptavidin-alkaline phosphatase and the substrate BCIP-NBT Plus (Mabtech). The spot number was enumerated and analyzed using the CTL ImmunoSpot reader and ImmunoSpot software (Cellular Technology Ltd., OH).

### Determination of anti-β-galactosidase IgG in serum

ELISA plates (Maxisorp, Nunc, Denmark) were coated with 50 µl/well of 1 µg/ml β-galactosidase (#10105031001, Roche Diagnostic GmBH, Mannheim, Germany) in 0.1 M carbonate buffer. The covered plates were incubated at 4°C over night. Next day, the plates were washed five times with PBS containing 0.05% Tween-20 (PBS-Tween), and blocked with 5% milk in PBS (100 µl/well) for 3 h at room temperature. Thereafter, mouse serum serially diluted from 1/200 to 1/204800 in PBS-Tween was added (50 µl/well) to the wells, and the plates were incubated at 4°C over night. The plates were again washed five times with PBS-Tween, and 50 µl horse radish peroxidase conjugated anti-mouse total IgG (Southern Biotech, Birmingham, AL) diluted 1/5000 in PBS-Tween was added to each well. After incubation for 3 h at room temperature, the plates were washed with PBS-Tween and 50 µl/well of OPD (P9187, Sigma-Aldrich) was added. The reaction was stopped after 7 min with 25 µl 1 M HCl. The absorbance was measured at 490 nm, using a VICTOR^2^ 1420 Multilabel Counter (PerkinElmer, Waltham, MA). Endpoint titers were calculated according to the method described by Frey [28] or at the dilution when the absorbance fell below 0.2.

### Intracellular cytokine staining

Splenocytes were stimulated with the SIINFEKL peptide (1 µg/ml) and GolgiPlug (BD Biosciences, Stockholm, Sweden) for 4 h. Surface and intracellular stainings were performed using the Cytofix/Cytoperm™ Fixation/Permeabilization Solution set (BD Biosciences) according to the manufacturer's instructions. Anti-CD8, IFN-γ, TNF and IL-2 antibodies were purchased from BD Biosciences. Samples were analyzed on a FACSCanto II cytometer (BD Biosciences) and the data were processed using FlowJo (Tree Star, Ashland, OR).

### Analyses of memory T cell subsets

Antibodies directed against CD8, CD27, CD62L (BD Biosciences), CD43 (BioLegend, Nordic Biosite, Täby, Sweden), and CD127 (eBioscience, AH Diagnostics ab, Skärholmen, Sweden), as well as an H-2Kb/SIINFEKL pentamer (Proimmune, Oxford, UK) were used for immunoflorescence staining and subsequent flow cytometry analysis. Nonspecific binding was blocked by adding FITC-conjugated rat anti-mouse CD16/CD32 antibody (BD Biosciences). FITC-conjugated rat anti-mouse CD4 and CD19 (BD Biosciences) were also used to exclude cells specific for these markers from the analysis. Samples were analyzed on a FACSCanto II cytometer (BD Biosciences) and the data were processed using FlowJo (Tree Star).

### Statistics

Statistical analyses were performed using GraphPad Prism version 5.02 for Windows, GraphPad Software (San Diego California, USA). The Mann-Whitney test was used for statistical comparisons. A p-value of 0.05 or less was considered as significant.

## Results

### Immunization constructs

Plasmids used for direct immunizations or as templates for *in vitro* transcription of RNA were constructed using standard cloning techniques. Four vector modalities with two different inserts were used in this study: *in vitro* transcribed mRNA, RREP (an *in vitro* transcribed SFV RNA replicon), DREP (a DNA-launched SFV replicon) and pCMV (a conventional DNA expression plasmid), as listed in Table 1. For the assessment of cellular immune responses a gene segment coding for the strong CD8+ T cell restricted MHC class I epitope derived from ovalbumin (SIINFEKL) was attached to the 3′ end of the luciferase gene (mRNA-tLuc, RREP-tLuc and DREP-tLuc). Constructs used to elicit a humoral immune response contained the *lacZ* gene encoding β-galactosidase (β-gal), a common reporter gene and inducer of antibody responses *in vivo*. All plasmids were sequenced and biochemically verified for expression *in vitro* (data not shown).

### Intradermal injection of replicon RNA induces a cellular immune response which is improved by electroporation

**Figure 1:**
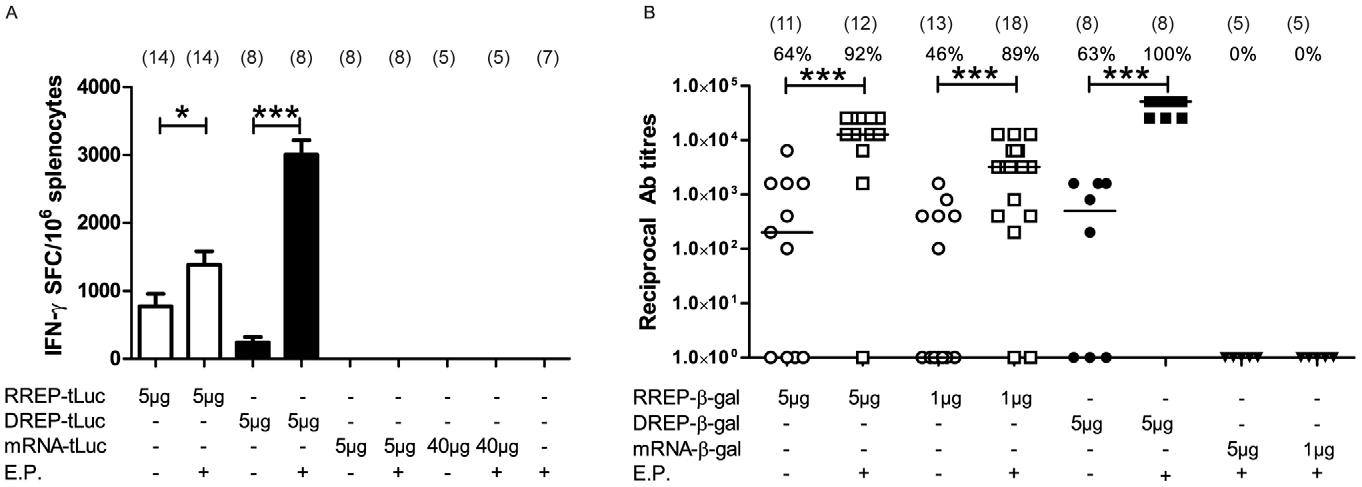
Immune responses after electroporation. (a) Antigen-specific IFN-γ positive CD8^+^ T cells per million splenocytes ten days after intradermal immunization either with or without electroporation. Electroporation significantly increases the number of positive cells in RREP-tLuc and DREP-tLuc immunized animals (p<0.05 and p<0.001 respectively). Data shows average number of positive cells from two separate experiments with error bars showing standard error of the mean. The total number of mice analyzed is indicated in parenthesis above each bar. (b) ELISA for total anti-β-galactosidase IgG antibody responses in sera from immunized mice 14 days post immunization. Electroporation significantly increases the antibody responses for both RREP-β-gal and DREP-β-gal (p<0.001). Data are plotted as reciprocal end-point titers and shown for each individual mouse. The total number of mice analyzed is indicated in parenthesis and the percentage of responding mice is indicated above each group. *Abbreviations*: SFC = spot forming cells, E.P. = electroporation, and Ab = antibody.

We first determined the immune response induced in mice after a single i.d. immunization with mRNA-tLuc, RREP-tLuc or DREP-tLuc with or without electroporation (E.P.). Ten days post immunization, mice were sacrificed and purified splenocytes were stimulated with the SIINFEKL epitope peptide and the number of antigen-specific interferon-γ (IFN-γ) producing CD8+ T cells was analyzed by ELISpot. I.d. injection of RREP-tLuc in combination with E.P. generated a robust response and resulted in an almost two-fold increase (p<0.05) in the number of IFN-γ producing spot forming CD8+ T cells as compared to non-E.P. mice. For DREP-tLuc, the positive effect of E.P. was even more pronounced and gave a 12-fold increase (p<0.001). Neither the naïve control group, nor the groups that received an i.d. immunization with 5 or 40 µg mRNA-tLuc developed any detectable specific immune responses (Figure 1a).

### Intradermal injection of replicon RNA induces a humoral immune response which is improved by electroporation

To investigate if E.P. also improved humoral immune responses to administered RNA replicons, mice were injected with RREP-β-gal (1 or 5 µg), DREP-β-gal (5 µg) or mRNA-β-gal (1 or 5 µg) i.d. with or without E.P. Fourteen days post immunization, antibody titers in sera from immunized mice were measured by ELISA. The anti-β-galactosidase IgG endpoint titers were significantly increased by E.P. (p<0.001) in groups immunized both with the higher and lower dose of RREP-β-gal and in the DREP-β-gal group (Figure 1b). However, no specific antibody response could be detected in groups immunized with mRNA-β-gal.

### Relative expression levels and cellular immune response

**Figure 2:**
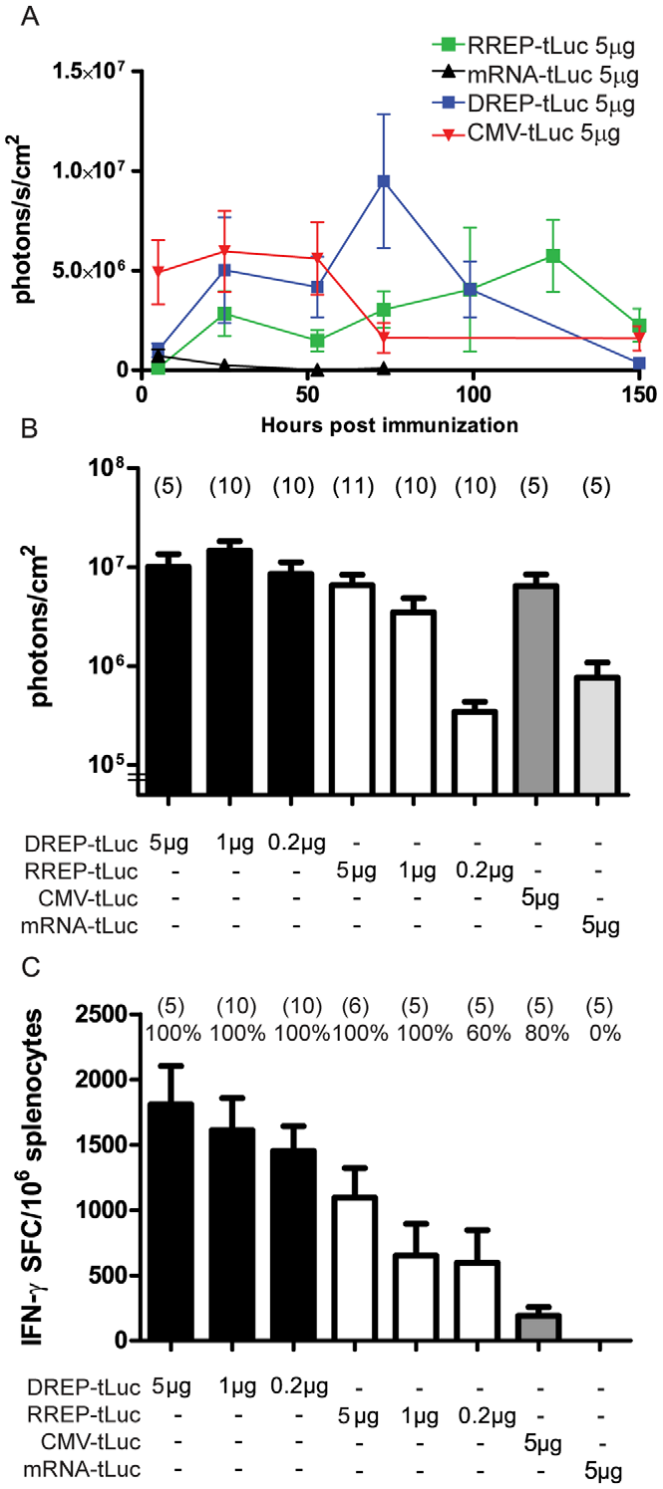
*In vivo* antigen expression and the immune response after electroporation. (a) Luciferase activity at different time points measured in photons/s/cm^2^. Data shows average activity for each group and error bars indicate SEM (number of mice per group indicated in Figure 2b). (b) Cumulative luciferase activity measured in photons/cm^2^. Data shows average accumulated luciferase activity up to 150 hours post immunization for each group (number of mice per group indicated in parentheses above each bar) with error bars showing standard error of the mean. (c) Antigen-specific IFN-γ positive CD8^+^ T cells per million splenocytes 10 days post-immunization. Data shows average number of positive cells with error bars showing standard error of the mean. The total number of mice analyzed is indicated in parenthesis and the percentage of responding mice is indicated above each group.

The amount of antigen produced *in vivo* from a specific vaccine vector may be of importance for the magnitude of the immune response. In order to correlate the induction of interferon-γ secreting CD8+ T cells to the relative amount of antigen produced *in vivo* by different RNA and DNA vectors, we measured the relative expression levels of several immunization constructs over time. Mice were immunized with luciferase-encoding RREP-tLuc (0.2, 1 or 5 µg), DREP-tLuc (0.2, 1 or 5 µg), mRNA-tLuc (5 µg) or pCMV-tLuc (5 µg) by i.d. injection followed by E.P. Luciferase activity was monitored repeatedly over a 6 day period using an *in vivo* imaging system (Figure 2a) and the accumulated luciferase production over the test period was calculated for each individual mouse. All immunizations resulted in *in vivo* luciferase activity, where the accumulated signal in DREP-tLuc, the two highest concentrations of RREP-tLuc and pCMV-tLuc immunized groups were within the same order of magnitude (Figure 2b). Mice immunized with the low dose of RREP-tLuc and mRNA-tLuc had a considerably lower accumulated luciferase activity. In the mRNA-tLuc immunized group this was due to a short-lived steadily declining luciferase expression, whereas the luciferase activity of all other groups initially increased for a few days and then declined towards the end of the experiment (Figure 2a). The CD8+ T cell responses in these animals were determined by ELISpot analysis 10 days post immunization. High numbers of antigen specific IFN-γ positive splenocytes were recorded for the high doses of DREP-tLuc and RREP-tLuc, while pCMV-tLuc produced a considerably lower response (Figure 2c). Again, mRNA-tLuc did not elicit any detectable specific immune response although a substantial luciferase activity had been recorded (Figure 2b).

### Intradermal injection of replicon RNA followed by electroporation induces a cellular memory immune response which is further increased by a booster immunization

**Figure 3:**
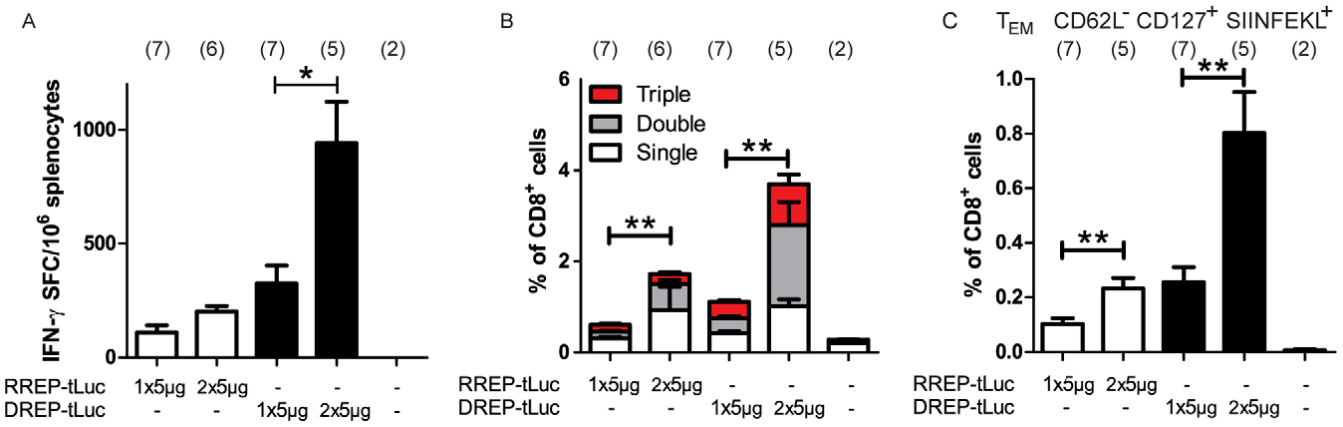
Cellular immune response 5 weeks post immunization. (a) Antigen-specific IFN-γ positive CD8^+^ T cells per million splenocytes, (b) Proportion responding CD8^+^ T cells as determined by intracellular staining of IFN-γ, IL-2 and TNF after SIINFEKL-peptide stimulation, or (c) proportion effector memory CD8^+^ T cells (pentamer H-2Kb/SIINFEKL positive CD8^+^CD62L^−^CD127^+^ cells) 5 weeks after the last intradermal immunization in combination with electroporation. Mice were either given one immunization (1×5 µg) or two immunizations 5 weeks apart (2×5 µg). Data shows average number of positive cells with error bars showing standard error of the mean. A booster immunization significantly increased the cellular memory response for both RREP-tLuc and DREP-tLuc (p<0.01). The total number of mice per group is indicated in parenthesis above each bar.

To investigate whether a cellular memory immune response could be induced by the replicons, mice were immunized with RREP-tLuc or DREP-tLuc in combination with E.P. Five weeks after the immunization, tLuc-immunized mice were sacrificed and subjected to ELISpot analysis as described above. In a parallel study aiming at showing the effect of a booster injection, mice were immunized twice five weeks apart and then sacrificed after five more weeks and subjected to ELISpot analysis. Both RREP-tLuc and DREP-tLuc elicited SIINFEKL-specific CD8+ T cells that remained during the contraction phase of the cellular immune response (Figure 3a). The booster immunization gave a statistically significant increase (p<0.05) of antigen-specific CD8+ T cells in animals immunized with DREP-tLuc, while we observed a trend of an increase of these cells in RREP-tLuc immunized mice (p = 0.0513). These results were confirmed by staining splenocytes for antigen specific CD8+ T cells using an H-2Kb/SIINFEKL pentamer (Figure S2a).

The induction and maintenance of polyfunctional CD8+ T cells contributes to effective antiviral immunity [29], [30], [31], [32]. Therefore we determined the presence of antigen-specific CD8+ T cells capable of producing one or more cytokines in response to stimulation with the SIINFEKL peptide by intracellular cytokine staining for IFN-γ, TNF and IL-2 followed by flow cytometry analysis (Figure 3b). The total number of responding cells increased significantly between the prime and the boost both for RREP and DREP immunized mice (p<0.01).

Splenocytes from these animals were further analyzed by staining antigen-specific CD8+ T cells for memory markers (CD127 and CD62L, Figure S2b). Both effector (T_EM_; CD127^+^CD62L^−^) and central memory CD8+ T (T_CM_; CD127^+^CD62L^+^) cell subsets [33] were found in all immunized mice. A booster immunization led to a significant increase of T_EM_ cells in both RREP-tLuc (p<0.005) and DREP-tLuc immunized animals (p<0.005), as shown in Figure 3c. Thus, a proportion of the remaining antigen-specific CD8+ T cells five weeks post immunization represents memory cells with a high effector capacity.

## Discussion

This study describes a new method of delivering naked Semliki Forest virus RNA replicons that promotes strong cellular and humoral immune responses. We have previously established that i.m. delivery of *in vitro* transcribed naked RNA-replicons (RREP) can elicit protective immunity *in vivo* [13]. Intradermal delivery of DREP also induces a potent immune response [12], and here we report that the same holds for naked RREP and that this response can be further improved by topical E.P. In contrast, i.d. mRNA-immunized mice failed to induce any detectable immune responses. Contrary to previously reported studies with mRNA vaccines that elicited immune responses, we have used a single immunization of naked RNA [4], [5], [34]. RREP and mRNA differ in many intrinsic properties which could explain their difference in immunogenicity. A key difference is that once inside a cell, RREP amplifies with subsequent high levels of antigen expression. This is in contrast to mRNA which gives a relatively much lower antigen expression. The stability differs between mRNA and RREP with mRNA generally having a very short half-life *in vivo* whereas alphavirus replicon RNA has a predicted highly ordered structure and is partially resistant to degradation [23]. In addition, alphavirus genomes have repetitive RNA elements in the 3′ untranslated region (UTR) that prevent deadenylation [35] by recruiting HuR, a cellular regulator of mRNA stability [36]. Efforts to improve stability, *in vivo* half-life and reporter-gene expression in conventional mRNA has been made by adding Venezuelan Equine Encephalitis virus-derived UTR [37]. Indeed, luciferase activity after mRNA-tLuc *in vivo* E.P. is decaying rapidly resulting in a substantial reduction of accumulated antigen expression as compared to the equivalent dose of RREP-tLuc (Figure 2a and 2b). The difference in antigen expression between the vectors is further underscored if one considers the difference in molar mass between mRNA-tLuc (∼0.7 MDa) and RREP-tLuc (∼3.0 MDa). However, interpreting the measured luciferase expression levels *in vivo* is not straightforward since the luminescence only reflects the steady state, i.e. how much functional luciferase that can be detected at the time of analysis. We do not know if the rate of luciferase degradation differs between mice immunized with the different vectors, hence we cannot completely accurately estimate the total amount of luciferase expressed. For example, replicon-containing vectors might via efficient induction of innate signaling attract more cytotoxic immune cells resulting in increased luciferase sequestration, thereby making us underestimate the total expression in RREP-tLuc relative to mRNA-tLuc immunized mice.

Another reason why RREP, but not mRNA, induces an immune response could be that cellular mRNAs lack several of the immunostimulatory properties that most viral RNA possess. Although single stranded mRNA vaccines activate TLR7 signaling [5], SFV replication turns on additional immune signaling from several PRR sensing various forms of RNA species present in endosomes and in the cytoplasm of the host cell such as TLR3, MDA5, PKR and, to a lesser extent, RIG-I [17], [18]. Subsequent downstream signaling results in type I interferon production that links innate and adaptive immune responses, and promotes both antibody production, CD4+ helper T-cell induction and cytotoxic immune responses. Replicon-based vectors are thus provided with inherent adjuvant properties. The adjuvant effect becomes evident when comparing expression levels and the resulting immune response between RREP-tLuc and pCMV-tLuc. Despite a higher accumulated luciferase activity in pCMV-tLuc, it still elicits a significantly lower immune response in our experimental system (Figure 2). In studies using conventional DNA vaccines, increased *in vitro* expression has translated into improved cellular immune responses [38]. Indeed, in direct comparisons DNA-launched alphavirus replicons have been shown to increase antigen expression *in vitro* as compared to conventional plasmid DNA vectors [12], [15], [20]. However, the difference in antigen expression has been small (two–five-fold) and does not solely account for the improved immunogenicity, since replicon DNA has induced similar immune responses at up to 600-fold lower doses than conventional DNA vaccines. Presented in this study is a direct comparison of antigen expression in the skin of mice over time, and it is evident from this data that the accumulated *in vivo* expression levels from a conventional DNA expression vector and a DNA-launched replicon are within the same order of magnitude. These results indicate that it is the vaccine modality, and not the actual antigen expression level, that is crucial for the immune response, and underline the importance of innate stimuli in order to form a strong immune response.

Comparing groups immunized i.d. with RREP to those immunized with DREP, immune responses were not statistically different (p = 0.1) without E.P. When E.P. was applied, both RREP and DREP responses increased, 2-fold and 12-fold, respectively. The reason why the DREP-induced responses increased proportionally more could potentially be because the double stranded DREP DNA is a larger molecule (∼8.0 MDa) as compared to the single stranded RREP RNA (∼3.0 MDa) and arguably has more difficulties entering an intact cellular membrane. In addition, DREP needs to enter the nucleus of the cell in order to transcribe the replicon, while RREP only needs to enter the cell cytoplasm. Possibly, electroporation efficiently opens pores also in the nuclear membrane and drives the DNA molecule into the nucleus.

RNA replicon-based vaccines offer a biosafe alternative to other gene-based vaccine technologies developed to date. It does not require a viral delivery vehicle nor does it require viral structural genes. Thus, the hypothetical possibility of reversion or gene conversion of the vector into a pathogenic phenotype is obviated. Moreover, RNA-based replicon vaccines cannot integrate into the host genome. Albeit several studies have failed to demonstrate integration of plasmid-based vaccines [39], [40], [41], [42], this still remains a theoretical risk that may cause regulatory authorities to delay commercial development of DNA vaccines. Also in the absence of genomic integration, both conventional and replicon-encoding plasmid DNA can persist in the tissue and, depending on the antigen, expression can be detected for months after injection [39], [41], [43], [44]. This raises additional concerns since persisting viral infections in humans, such as HIV or hepatitis C virus, are associated with dysfunctional CTL responses [45], [46]. Correspondingly, persisting antigen from recombinant Adenovirus vectors capable of high level antigen expression have been shown to cause tolerization rather than a functional immune response in certain cases [47].

In summary, intradermal administration of Semliki Forest virus RNA replicons in combination with topical electroporation offer a non-invasive, biosafe alternative that can be used not only in prophylactic vaccination strategies, but also in therapeutic settings.

## Supporting Information

Figure S1 **Calculation of accumulated luciferase expression** ***in vivo*** **.** This figure shows a test data-set used to validate the formula used to calculate the accumulated luciferase signal (Figure 2b). To calculate the integrated luciferase signal between two time points (area under the curve) the following formula was used: I = (t_2_−t_1_)(s_2_+(s_1_−s_2_)/2) where I is the integrated signal, t_1_ is timepoint 1, t_2_ timepoint 2, s_1_ signal at timepoint 1 and s_2_ signal at timepoint 2.
(TIF)

Figure S2 **Analyses of memory T cell subsets.** The induction of different SIINFEKL-specific CD8^+^ memory T cell subsets was determined by CD127, CD62L, CD43 and CD27 staining 5 weeks after the last immunization. In accordance with the results from the IFN-γ ELISPOTs, SIINFEKL^+^CD8^+^ cells were increased or slightly increased after boost with DREP-tLuc or RREP-tLuc, respectively (p = 0.0101 or p = 0.0513, a). Central memory (T_CM_; CD127^+^CD62L^+^, b) CD8^+^ T cells were present in the spleens of immunized mice. However, there were no statistically significant differences in the proportions of these cells between primed and boosted animals, neither for RREP-tLuc or DREP-tLuc. In addition, the presence of SIINFEKL-specific CD8^+^ T cell subsets with a high recall capacity (CD27^+^CD43^−^, c) was demonstrated, but with no statistically significant differences between primed and boosted animals (c).
(TIF)
