# Supplementary figures and images for: Intradermal Electroporation of Naked Replicon RNA Elicits Strong Immune Responses
Source: PLoS One. 2012 Jan 4;7(1):e29732. doi: 10.1371/journal.pone.0029732 (PMC3251598; doi:10.1371/journal.pone.0029732)

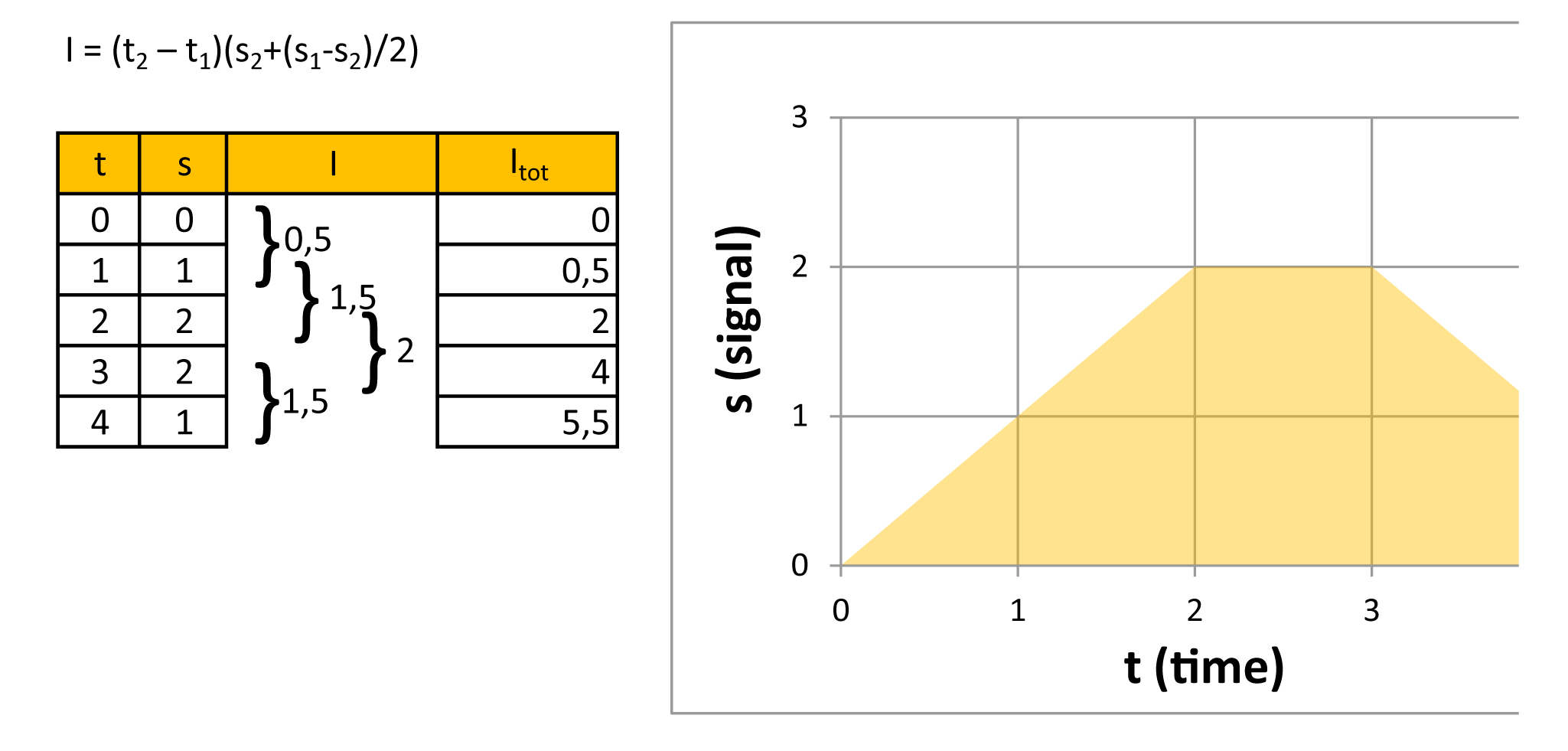

Supplement: Figure S1 — Calculation of accumulated luciferase expression in vivo . This figure shows a test data-set used to validate the formula used to calculate the accumulated luciferase signal (Figure 2b). To calculate the integrated luciferase signal between two time points (area under the curve) the following formula was used: I = (t2−t1)(s2+(s1−s2)/2) where I is the integrated signal, t1 is timepoint 1, t2 timepoint 2, s1 signal at timepoint 1 and s2 signal at timepoint 2. (TIF) [file pone.0029732.s001.tif]

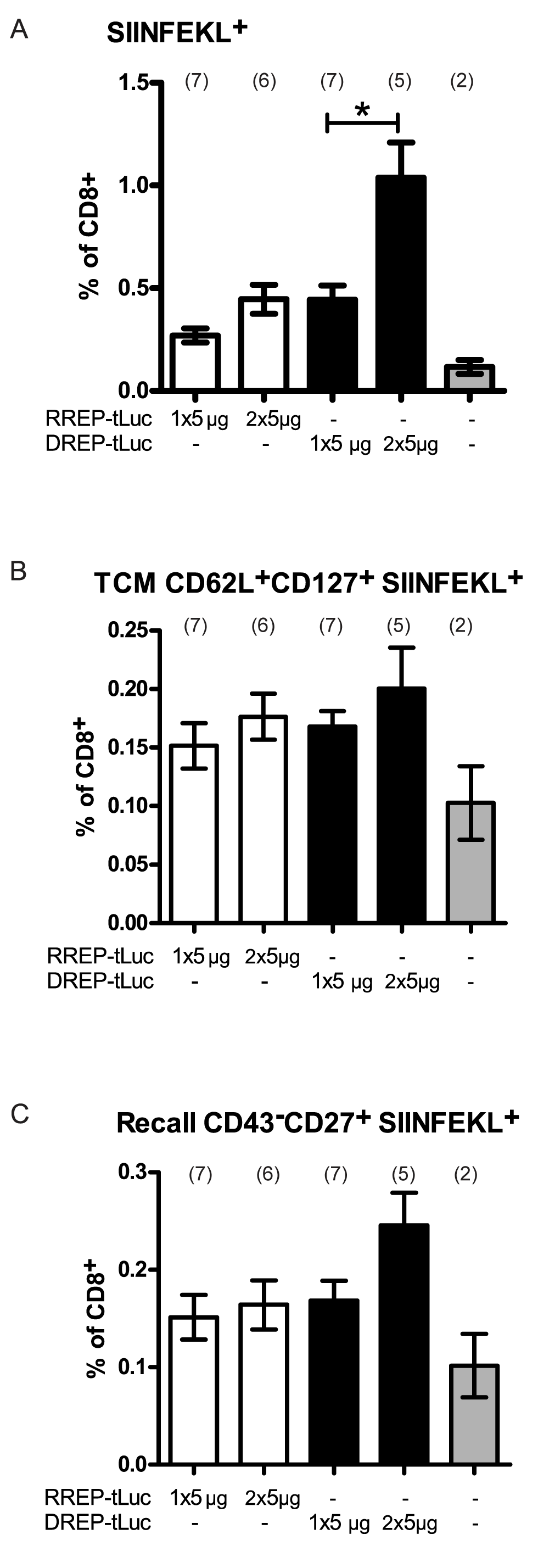

Supplement: Figure S2 — Analyses of memory T cell subsets. The induction of different SIINFEKL-specific CD8+ memory T cell subsets was determined by CD127, CD62L, CD43 and CD27 staining 5 weeks after the last immunization. In accordance with the results from the IFN-γ ELISPOTs, SIINFEKL+CD8+ cells were increased or slightly increased after boost with DREP-tLuc or RREP-tLuc, respectively (p = 0.0101 or p = 0.0513, a). Central memory (TCM; CD127+CD62L+, b) CD8+ T cells were present in the spleens of immunized mice. However, there were no statistically significant differences in the proportions of these cells between primed and boosted animals, neither for RREP-tLuc or DREP-tLuc. In addition, the presence of SIINFEKL-specific CD8+ T cell subsets with a high recall capacity (CD27+CD43−, c) was demonstrated, but with no statistically significant differences between primed and boosted animals (c). (TIF) [file pone.0029732.s002.tif]
